# Association Between Vitamin D Receptor BsmI Polymorphism and Low Bone Mineral Density in Postmenopausal Women in the MENA Region

**DOI:** 10.3390/pathophysiology32010006

**Published:** 2025-02-01

**Authors:** Tara Al-Barazenji, Asma Allouch, Nedhal Al Husaini, Sondos Yousef, Wisam Nabeel Ibrahim, Amal Al-Haidose, Hatem Zayed, Atiyeh M. Abdallah

**Affiliations:** Department of Biomedical Sciences, College of Health Sciences, QU Health, Qatar University, Doha 2713, Qatar; ta1706200@student.qu.edu.qa (T.A.-B.); aa1602752@student.qu.edu.qa (A.A.); na2201028@student.qu.edu.qa (N.A.H.); sy1701619@student.qu.edu.qa (S.Y.); w.ibrahim@qu.edu.qa (W.N.I.); amalah@qu.edu.qa (A.A.-H.); hatem.zayed@qu.edu.qa (H.Z.)

**Keywords:** vitamin D receptor, BsmI polymorphism, ApaI polymorphism, TaqI polymorphism, low BMD, osteoporosis and osteopenia, postmenopausal, meta-analysis

## Abstract

Background/Objectives: Low bone mineral density increases the risk of bone fractures, and this condition is especially common in postmenopausal women. Genetic factors significantly influence bone mineral density. This meta-analysis examined the relationship between vitamin D receptor (VDR) gene polymorphisms (BsmI, ApaI, and TaqI) and bone mineral density in postmenopausal women in the Middle East and North Africa (MENA) region. Methods: The PubMed, Embase, Scopus, and Web of Science databases were searched from inception to March 2024 for case–control studies on VDR BsmI, ApaI, and TaqI polymorphisms and their relationship with low bone density. Associations with low bone mineral density were tested with respect to different genetic models (dominant, recessive, allelic) using RevMan v5.3. Results: The meta-analysis included seven studies for BsmI, six for ApaI, and seven for TaqI, representing 704/689 cases/controls for BsmI, 914/711 for ApaI, and 974/895 for TaqI. No significant association was found between VDR polymorphisms and low bone mineral density in postmenopausal women, except in the dominant model (CC + CG vs. GG) for the BsmI variant (OR = 1.27, 95% CI: 1.01–1.59, *p* = 0.04). Conclusions: We found a modest association between the BsmI polymorphism and increased risk of low bone mineral density (BMD) in postmenopausal women from the MENA region, suggesting its potential as a biomarker. No associations were observed for ApaI or TaqI. These findings highlight the complex genetic–environmental interactions influencing BMD.

## 1. Introduction

Osteopenia and osteoporosis, characterized by low bone mineral density (BMD) and microarchitectural deterioration of bone tissue, significantly increase fracture risk, particularly in postmenopausal women [1]. In the Middle East and North Africa (MENA) region, the prevalence of low BMD is increasing, primarily due to aging populations, sedentary lifestyles, and a lack of awareness about bone health [2]. Studies have shown that the number of deaths and disability-adjusted life years DALYs attributable to low BMD had almost doubled in the region from 1990 to 2019 [3]. Postmenopausal women face a unique set of challenges with respect to bone health, not least the hormonal changes associated with menopause. The decrease in estrogen production by the ovaries during menopause deprives the body of a multifunctional hormone that maintains bone density through its actions on osteoblasts (bone-forming cells) and osteoclasts (bone-resorbing cells) [4]. Appropriate levels of estrogen suppress osteoclast activity, slowing down bone resorption [5]. Consequently, when estrogen levels drop during menopause, bone resorption increases, accelerating the loss of bone density. This process can result in porous, fragile bones and increase the risk of fractures, particularly in weight-bearing areas such as the hip, spine, and wrist [5].

The decrease in estrogen levels also has adverse consequences on calcium homeostasis and overall bone metabolism. Calcium is a crucial mineral for bone health, as it provides structural support that maintains bone strength and integrity. A decrease in estrogen alters calcium homeostasis and, in response, the body may compensate by utilizing calcium from the bones, further contributing to bone density loss. Additionally, changes in bone metabolism can alter bone turnover and remodeling, increasing the risk of fracture. These complex and interrelated factors explain why postmenopausal women are particularly vulnerable to bone density loss and emphasize the importance of monitoring and managing bone health during this life stage [6,7].

Genetic factors also impact bone density. Of particular interest is the vitamin D receptor (*VDR*) gene [8], which plays a crucial role in regulating calcium balance and bone remodeling by controlling the systemic effects of vitamin D. Research has shown a significant link between polymorphisms in the VDR gene and reduced BMD [9]. Over 900 allelic variants of the VDR gene have been identified. Some of these variants reduce the receptor’s ability to bind calcitriol effectively, disrupting vitamin D and calcium homeostasis [10]. Several of these polymorphisms, including BsmI (rs1544410 G>A), ApaI (rs7975232 C>A), and TaqI (rs731236 T>C) have been associated with changes in bone mineral density and a greater risk of bone fractures [11].

The TaqI polymorphism (rs731236) is situated in exon 9, near the 3′ untranslated region (3′-UTR) of the VDR gene at the chromosomal location of chr12:47845446, within the noncoding region. It encodes a silent mutation resulting from a substitution of thymine (T) with cytosine (C). Despite being silent, this SNP has the potential to alter certain functional properties of the protein [12]. Similarly, the BsmI polymorphism (rs1544410 G>A) with the chromosomal location chr12:47845480 is in intron 8, encoding a substitution of guanine (G) to adenine (A) [9], and ApaI polymorphism (rs7975232), with the location of chr12:47843088, is situated in intron 8, encoding a substitution of C to A. Both BsmI (rs1544410 G>A) and ApaI (rs7975232 C>A) are restriction site polymorphisms. These variants were studied in association with low bone mineral density due to their potential influence on VDR function, and consequently calcium metabolism and bone health [13].

*VDR* is therefore an important focus as a biomarker and therapy target, especially in the context of postmenopausal low BMD [14,15], and understanding how different *VDR* polymorphisms affect BMD risk would be helpful for informing healthcare strategies and preventive measures [16].

Several studies have illustrated an association between these polymorphisms and the risk of low bone mineral density in many different populations. Yet, the association in data from the MENA population was not explicitly clear. We therefore conducted a meta-analysis to comprehensively examine the literature on VDR polymorphisms BsmI (rs1544410 G>A), ApaI (rs7975232 C>A), and TaqI (rs731236 T>C), and their potential impact on bone health in postmenopausal women in the MENA region [17,18]. The findings of this meta-analysis not only contribute to our understanding of the genetic determinants of low bone density in postmenopausal women within the MENA region, but also provide valuable insights for clinicians, healthcare policymakers, and researchers seeking to develop targeted interventions and prevention strategies to prevent the adverse consequences of age-related changes in bone health.

## 2. Materials and Methods

### 2.1. Search Strategy

This review followed Preferred Reporting Items for Systematic Review and Meta-Analysis (PRISMA) guidelines (Table S1) [19]. PubMed, Embase, Scopus, and Web of Science databases were searched from the date of inception to 15 October 2023 using the following terms: [(bone density OR Low bone mineral density OR osteoporosis OR osteopenia) AND (post menopause OR post-menopausal) AND (gene OR genotype OR polymorphism OR SNP) AND (algeria OR egypt OR libya OR morocco OR tunisia OR south sudan OR sudan OR middle east OR bahrain OR iraq OR jordan OR kuwait OR lebanon OR oman OR qatar OR saudi arabia OR syria OR united arab emirates OR yemen OR somalia OR mauritania OR iran OR turkey OR arab)]. MeSH terms were used when applicable to the database. Four authors individually screened each article by title and abstract and then evaluated the full text to fully assess eligibility for inclusion. Furthermore, the reference lists of the retrieved articles were reviewed by title to ensure a comprehensive search. This study is registered at https://doi.org/10.17605/OSF.IO/CJ2UG.

### 2.2. Inclusion and Exclusion Criteria

This study was designed according to a PICOS strategy: population, postmenopausal women from the MENA region; intervention, association between BsmI (rs1544410 G>A), ApaI (rs7975232 C>A), and TaqI (rs731236 T>C) variants and low BMD; and primary outcome and the presence or absence of an association (Table 1). The inclusion criteria were as follows: (1) low BMD conditions such as osteoporosis and osteopenia; (2) a study population ethnicity from the MENA region, including the 22 Arab countries, Turkey, and Iran; (3) the study design was a case–control or nested case–control design; (4) the cases were strictly defined as postmenopausal women; (5) *VDR* gene polymorphisms (BsmI, ApaI, and TaqI) were genotyped and evaluated with respect to the risk of low BMD; and (6) genotype frequencies for *VDR* gene polymorphisms (BsmI, ApaI, and TaqI) were included for cases and controls. The exclusion criteria were as follows: (1) the study population included other ethnicities; (2) the case definition lacked postmenopausal women; (3) genotype frequency data were omitted from full-text articles and Supplementary Materials; or (4) the study was a duplicate.

### 2.3. Data Extraction

Four independent investigators extracted data from eligible studies. The extracted characteristics included the first author, publication year, region of study, study population ethnicity, age range, total number of cases and controls, genotypes analyzed, and genotyping method. Any disagreements in evaluations were carefully reviewed and re-evaluated through a structured discussion process, involving all evaluators. This iterative approach ensured that differing perspectives were considered, and a thorough consensus was reached, reflecting a mutually agreed-upon judgment. The extracted data were analyzed by four investigators, and the most common polymorphisms (*VDR* BsmI, TaqI, ApaI) were selected for meta-analysis.

### 2.4. Low BMD Definition

In this analysis, we included all patients with low BMD, encompassing those diagnosed with osteoporosis and osteopenia, in a combined analysis. This approach was necessitated by the limited number of studies and sample sizes available, which precluded the feasibility of performing separate subgroup analyses for osteoporosis and osteopenia. This approach was described by many researchers [20].

### 2.5. Quality Assessment

The Newcastle–Ottawa quality assessment scale (NOS) was used for quality assessment, which focuses on study group selection, the ability to compare the groups, and the advancement of exposure. Each criterion was assessed by answering, scoring, and summing specific questions. The Hardy–Weinberg equilibrium (HWE) was considered when we assessed each study.

### 2.6. Statistical Analysis

Heterogeneity between studies was assessed with the I^2^ statistic, which quantifies the proportion of total variation across studies that is attributable to heterogeneity rather than chance. This measure provides an indication of the consistency of results across the included studies. The pooled odds ratios (ORs) with 95% confidence intervals (CIs) in the forest plot were analyzed using a fixed-effects model (restricted maximum likelihood (REML) method). Begg’s funnel plot was used to qualitatively assess the risk of publication bias. All analyses were performed using RevMan v5.3. A *p*-value < 0.05 (two-sided) was considered statistically significant. Power analysis for the total population size was performed using the QUANTO program. The total number of cases and controls for each polymorphism was adequate to achieve an 80% study power at the significance level of 0.05.

## 3. Results

### 3.1. Search Outcome

**Figure 1:**
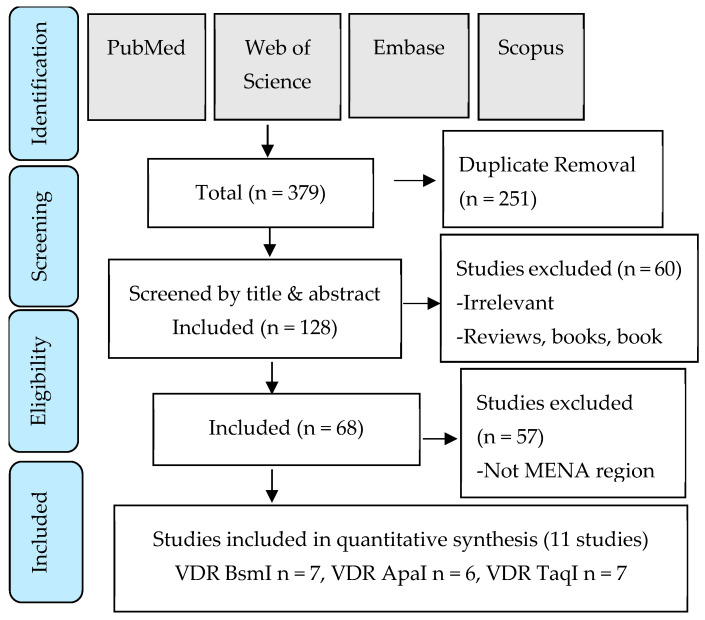
PRISMA flow chart detailing the search strategy and study selection process.

The literature screening and article selection process is shown in the PRISMA flow chart (Figure 1). The search strategy was devised to capture all genetic polymorphisms studied in association with low BMD in postmenopausal women from the MENA region. Eleven studies were enrolled for qualitative analysis, representing 1271 low-bone-density postmenopausal women and 1083 controls. The main characteristics of the selected studies are shown in Table 2.

### 3.2. Quality Assessment of Included Studies

According to the NOS quality assessment of case–control studies, all the selected articles met the quality requirements, each scoring ≥5 (Table 2). Therefore, none of the eligible articles were excluded. The Hardy–Weinberg equilibrium (HWE) is a principle used to determine if allele and genotype frequencies in a population remain constant from generation to generation in the absence of evolutionary influences. Among the included studies, one out of seven investigating the TaqI (rs731236 T>C) polymorphism did not adhere to HWE, while four out of seven studies on BsmI (rs1544410 G>A) variants showed deviations from it. For the ApaI (rs7975232 C>A) polymorphism, half of the studies were consistent with HWE.

### 3.3. Pooled Analyses

**Figure 2:**
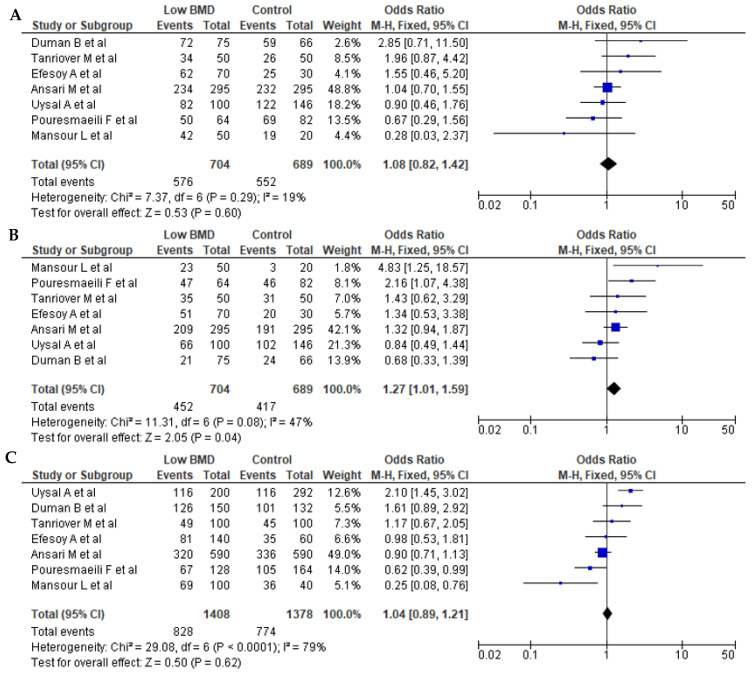
Forest plots of the BsmI (rs1544410 G>A) polymorphism. (**A**) Dominant, (**B**) recessive, and (**C**) allelic models. The *x*-axis represents the odds ratio or risk difference, and the *y*-axis lists the included studies. Each horizontal line represents a study’s confidence interval, with the square size proportional to its weight in the meta-analysis. The diamond at the bottom illustrates the pooled effect estimate and its 95% confidence interval, summarizing the overall association. This data was generated using these references: Uysal et al., 2008 [22], Tanriover et al., 2010 [23], Ansari et al., 2021 [25], Mansour et al., 2010 [26], Pouresmaeili et al., 2013 [28], Duman et al., 2004 [29], and Efesoy et al., 2011 [30].

**Figure 3:**
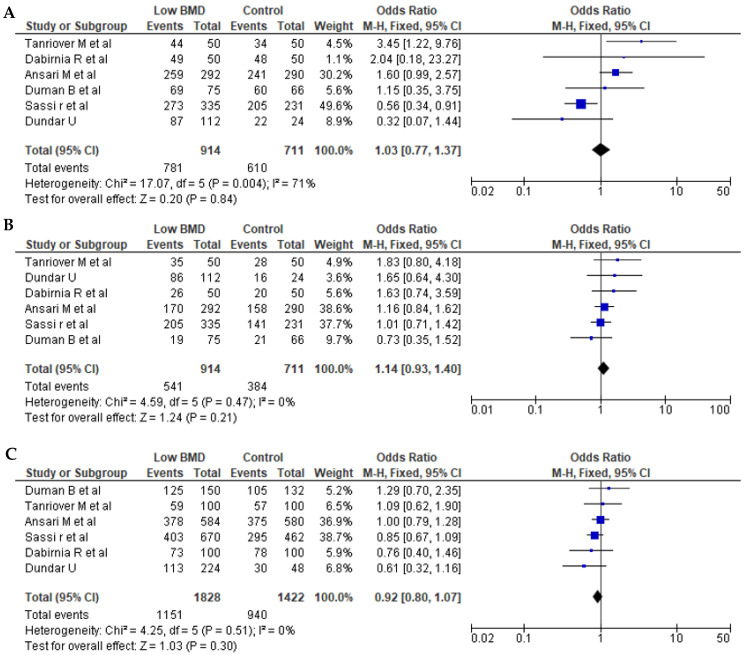
Forest plots of the ApaI (rs7975232 C>A) polymorphism. (**A**) Dominant, (**B**) recessive, and (**C**) allelic models. The *x*-axis represents the odds ratio or risk difference, and the *y*-axis lists the included studies. Each horizontal line represents a study’s confidence interval, with the square size proportional to its weight in the meta-analysis. The diamond at the bottom illustrates the pooled effect estimate and its 95% confidence interval, summarizing the overall association. This data was generated using these references: Sassi et al., 2015 [21], Tanriover et al., 2010 [23], Dundar et al., 2009 [24], Ansari et al., 2021 [25], Dabirnia et al., 2016 [27], and Duman et al., 2004 [29].

**Figure 4:**
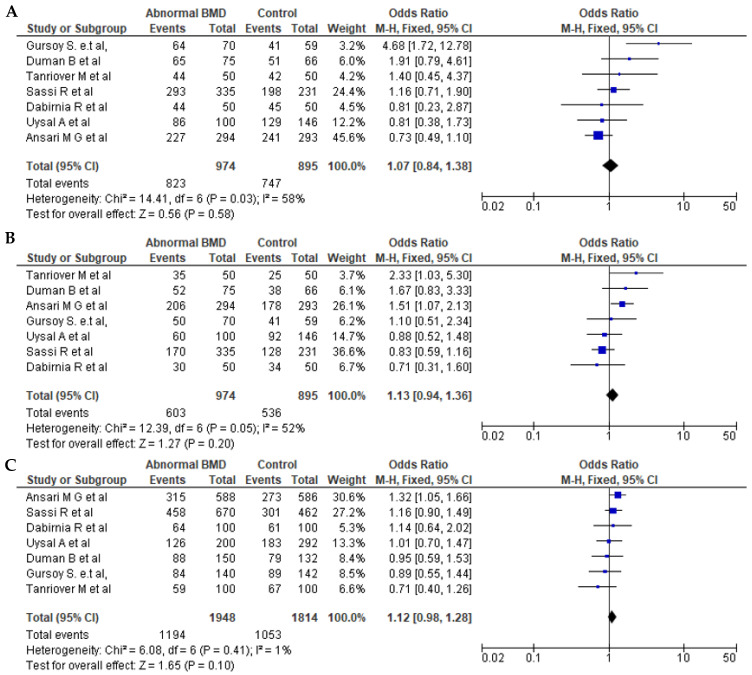
Forest plots of the TaqI (rs731236 T>C) polymorphism. (**A**) Dominant, (**B**) recessive, and (**C**) allelic models. The *x*-axis represents the odds ratio or risk difference, and the *y*-axis lists the included studies. Each horizontal line represents a study’s confidence interval, with the square size proportional to its weight in the meta-analysis. The diamond at the bottom illustrates the pooled effect estimate and its 95% confidence interval, summarizing the overall association. This data was generated using these references: Sassi et al., 2015 [21], Uysal et al., 2008 [22], Tanriover et al., 2010 [23], Ansari et al., 2021 [25], Dabirnia et al., 2016 [27], Duman et al., 2004 [29], and Gursoy et al., 2008 [31].

Seven articles for BsmI (rs1544410 G>A), six articles for ApaI (rs7975232 C>A), and seven articles for TaqI (rs731236 T>C) were included in pooled analyses. The genotypes and allele frequencies of BsmI, ApaI, and TaqI *VDR* gene polymorphisms in postmenopausal women with low BMD cases and controls are shown in Table 3. There were 704/689 cases/controls for BsmI, 914/711 cases/controls for ApqI, and 974/895 cases/controls for TaqI. A summary of the meta-analysis is shown in Table 4. For all *VDR* polymorphisms, there was no significant association between the presence of any of the genetic models with the prevalence of low BMD in postmenopausal women, except for the dominant model (AA + AG vs. GG) for BsmI (OR = 1.27, 95% CI: 1.01–1.59; *p* = 0.04). Figure 2, Figure 3 and Figure 4 show forest plots for BsmI, ApaI, and TaqI polymorphisms in dominant, recessive, and allelic models.

### 3.4. Publication Bias

**Figure 5:**
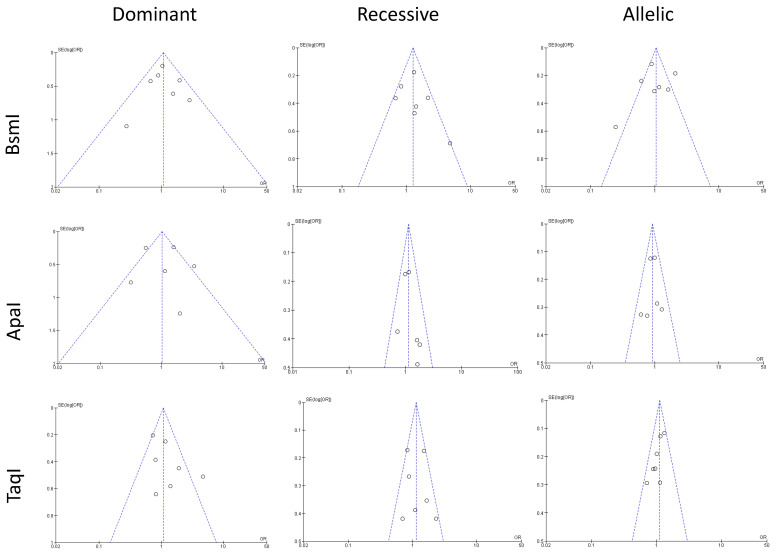
The figure shows funnel plots assessing publication bias for studies grouped by genetic polymorphisms of TaqI, BsmI, ApaI. The *x*-axis represents the effect size, and the *y*-axis shows the standard error. The central vertical line indicates the pooled effect estimate, while dashed lines outline the 95% confidence interval. Each circle represents an individual study. Symmetry suggests minimal bias, while asymmetry indicates potential bias or heterogeneity.

Publication bias was assessed using Begg’s funnel plot and Egger’s test for all three polymorphisms (Figure 5). BsmI (rs1544410 G>A), ApaI (rs7975232 C>A), and TaqI (rs731236 T>C) polymorphism funnel plots showed no evidence of publication bias for the dominant model, recessive model, or allelic model.

## 4. Discussion

Low BMD is a common disorder that increases the risk of fractures. The vitamin D receptor plays an important role in bone metabolism, and polymorphisms in the *VDR* gene have been associated with low BMD. The main aim of this meta-analysis was to review and assess whether *VDR* gene polymorphisms [BsmI (rs1544410 G>A), ApaI (rs7975232 C>A), TaqI (rs731236 T>C)] are associated with low BMD in postmenopausal women in the MENA region.

Seven studies were available, representing 704 cases and 689 controls, to analyze the association between the BsmI (rs1544410 G>A) polymorphism and low BMD. We detected a statistically significant association between BsmI and the risk of low BMD in the dominant model, but not in the recessive and allelic models. The BsmI polymorphism is in the 3′ untranslated region, which regulates gene expression, mRNA stability, and localization.

Seven studies were also available to examine associations between the TaqI (rs731236 T>C) polymorphism and low-BMD risk, with a total of 974 cases and 907 controls. However, there was no significant association for the allelic, dominant, or recessive models. With respect to the ApaI (rs7975232 C>A) polymorphism, six studies were comprehensively reviewed, with 914 cases and 711 controls, and with no significant impact on low BMD for any of the three models.

We observed a statistically significant association between the BsmI polymorphism and risk of postmenopausal low BMD (*p* < 0.05). The results on VDR BsmI polymorphisms and low BMD are inconsistent, possibly due to study data heterogeneity, or perhaps due to heterogeneity in the study data. A recently published meta-analysis identified the BsmI polymorphism as a susceptibility gene for postmenopausal low BMD in White populations but not Asian populations [32], and an older meta-analysis established a link between BsmI and postmenopausal low BMD in Chinese women [33]. Other meta-analyses suggest BsmI may protect against osteoporosis, while others report no association with postmenopausal low BMD in various populations, including Caucasian and Asian populations [34,35,36,37,38]. However, several other meta-analyses suggest the opposite, showing a lack of association with postmenopausal low BMD in an Asian population [39], White and Asian postmenopausal women [40], and postmenopausal women in general [41]. Similarly, other meta-analyses report a lack of an association with osteoporosis in the general Han Chinese population [42,43] and the overall population [44,45,46].

The ApaI (rs7312366 C>A) *VDR* polymorphism is reported to be associated with osteoporosis in postmenopausal women [47], with the polymorphism potentially influencing VDR function and consequently calcium metabolism and bone health. The results are conflicting with regard to the association between ApaI polymorphisms and BMD [48]. We found no significant association between any of the three ApaI models (dominant, recessive, and allelic) and BMD, suggesting that ApaI polymorphisms are not a major risk factor for low BMD in postmenopausal women.

We also found no significant association between the TaqI (rs731236 T>C) polymorphism and low bone mineral density in postmenopausal women in all three models (dominant, recessive, and allelic). Indeed, a study conducted in Spanish postmenopausal women [49] similarly found no association between this variant and low BMD, but a study in a group of Belarusian and Lithuanian postmenopausal women found that the presence of the TaqI C allele was a significant risk factor for low BMD [50]. Likewise, a meta-analysis of White and Asian cohorts revealed a strong association between TaqI polymorphisms and the risk of developing low BMD in White, but not Asian, individuals [51]. These conflicting results may be due to the risk conferred by multiple genes and environmental factors [52]. Moreover, the findings of genetic association studies are heavily influenced by sample size, and the small sample sizes of the studies included in this meta-analysis may result in a weak power to detect an association between the variant and low bone mineral density.

Biological factors may also explain the discordant findings. For instance, although the TaqI variant is in the coding sequence, it does not alter the amino acid sequence of the encoded protein. It is therefore considered a non-functional allele that can be used as a marker allele in association studies, where any detected association is due to nearby functional alleles. The strength of the linkage disequilibrium explains the association between functional and marker alleles, and can therefore explain differences in study findings [53].

Our study has some limitations. Most study sample sizes were small, and we excluded many studies that did not specify the target population as postmenopausal women, limiting the analysis. Furthermore, several studies deviated from HWE, though they were still included in the analysis. Deviations from HWE may suggest potential issues with the genetic data. While the studies included in this meta-analysis did deviate from HWE, we acknowledge that these deviations may affect the reliability and accuracy of the results. Future studies should carefully assess and correct for HWE deviations to ensure accurate genetic association analyses. Most studies were from Turkey and Iran, emphasizing a need for more comprehensive studies in other MENA countries. Moreover, there is no comprehensive publicly accessible registry for low BMD in the MENA region to facilitate data collection. Finally, we used QUANTO for power calculation, which has its own limitations for meta-analysis studies, as it does not account for heterogeneity.

Despite modeling data from several studies, our meta-analysis indicates that *VDR* polymorphisms may not be reliable markers for low-BMD risk in postmenopausal women in the MENA region. The lack of consistent associations between ApaI and TaqI polymorphisms and BMD suggests that these genetic variations alone are not significant predictors of low BMD. This finding highlights the complexity of low BMD, which likely results from the interplay of multiple genetic factors and environmental influences rather than single-gene polymorphisms. The inconsistency in the results across different studies and populations highlights the need to consider a broader range of genetic markers and larger, more diverse cohorts to better understand the genetic basis of low BMD. Our findings suggest that focusing only on *VDR* polymorphisms may overlook other critical genetic and non-genetic factors that contribute to low-BMD risk in postmenopausal women. This meta-analysis also emphasizes the need for comprehensive genetic screening and multifactorial approaches in assessing low-BMD risk and developing targeted prevention strategies. To further explore the complex relationship between VDR polymorphisms and low BMD, future studies should include larger, more diverse cohorts from across the MENA region to capture genetic variability and reduce the impact of potential genetic homogeneity in specific populations. Comprehensive genetic screening encompassing additional polymorphisms and other candidate genes, coupled with environmental and lifestyle data, is necessary to better understand the multifactorial etiology of low BMD.

## 5. Conclusions

We found a modest association between the BsmI (rs1544410 G>A) polymorphism and risk of postmenopausal low BMD, suggesting its potential role as a biomarker for susceptibility in this population. We detected no associations between ApaI (rs7975232 C>A) and TaqI (rs731236 T>C) and the risk of postmenopausal low BMD. The relationship between VDR polymorphisms and the risk of postmenopausal low BMD remains uncertain, and our findings suggest complex genetic–environmental interactions in the etiology of low BMD. Our findings indicate that genetic screening for BsmI may help identify postmenopausal women at higher risk of low BMD, though further validation is needed. Research should also focus on expanding studies in the MENA region to capture genetic variability and environmental factors and refine prevention strategies specific to this population.

## Figures and Tables

**Table 1:** Inclusion and exclusion criteria according to the Population, Intervention, Comparator, Outcomes, and Study design (PICOS) statement.

Categry	Included	Excluded
Population	MENA region	Other populations
Interventions	None	None
Comparators	Low bone-density postmenopausal women with *VDR* polymorphism genotypes (BsmI, ApaI, or TaqI)	-Articles with no genetic analysis -Articles with non-postmenopausal women -Articles with postmenopausal women with low bone density and another disease
Study design	Prospective, retrospective cohort, case reports, and research articles	-Reviews, books, protocols, guidelines, and animal studies
Primary outcome	Genetic variants reported in postmenopausal women with low bone density	Irrelevant
Secondary outcomes	Clinical phenotype variability in postmenopausal women with low bone density	Irrelevant

**Table 2:** Characteristics of studies included in the meta-analysis.

Study No.	Reference	Country	Ethnicity	Method	NOS	BMD Type	ApaI (rs7975232 C>A)	BsmI (rs1544410 G>A)	TaqI (rs731236 T>C)	Other Notes
1	Sassi et al., 2015 [21]	Tunisian	Arab	RFLP-PCR	5/7	Osteopenia	associated	-	associated	The presence of both ApaI and TaqI increases the risk
2	Uysal et al., 2008 [22]	Turkey	Turkish	RFLP-PCR	5/7	Osteoporosis	-	associated	-	The presence of both ApaI and TaqI increases the risk
3	Tanriover et al., 2010 [23]	Turkey	Turkish	RFLP-PCR	6/7	Osteoporosis	-	associated	-	
4	Dundar et al., 2009 [24]	Turkey	Turkish	RFLP-PCR	5/7	Osteoporosis	associated	-	-	
5	Ansari et al., 2021 [25]	KSA	Arab	TaqMan	6/7	Osteoporosis	associated	associated	associated	
6	Mansour et al., 2010 [26]	Egypt	Arab	RFLP-PCR	5/7	Osteoporosis	-	associated	-	
7	Dabirnia et al., 2016 [27]	Iran	Iranian	RFLP-PCR	6/7	Osteoporosis	-		-	
8	Pouresmaeili et al., 2013 [28]	Iran	Iranian	RFLP-PCR	5/7	Osteoporosis	-	associated	-	
9	Duman et al., 2004 [29]	Turkey	Turkish	RFLP-PCR	5/7	Osteoporosis	-	-	associated	
10	Efesoy et al., 2011 [30]	Turkey	Turkish	SSOP	5/7	Osteoporosis	-	associated	-	
11	Gursoy et al., 2008 [31]	Turkey	Turkish	RFLP-PCR	4/7	Osteoporosis	-	-	associated	

Abbreviations: NOS; Newcastle–Ottawa Score; BMD; bone mineral density, SSOP; sequence-specific oligonucleotide probes, RFLP; Restriction Fragment Length Polymorphism.

**Table 3:** Genotypes and allele frequencies of BsmI, ApaI, and TaqI VDR gene polymorphisms in low-BMD postmenopausal women cases, controls, and sample sizes.

Study	Cases					Control					Sample Size		HWE
	MM	MW	WW	M	W	MM	MW	WW	M	W	Cases	Control	
BsmI (rs1544410 G>A)													
Uysal et al., 2008 [22]	34	48	18	116	84	44	78	24	166	126	100	146	0.28
Tanriover et al., 2010 [23]	15	19	16	49	51	19	7	24	45	55	50	50	0.00
Ansari et al., 2021 [25]	86	148	61	320	270	104	128	63	336	254	295	295	0.05
Mansour et al., 2010 [26]	27	15	8	69	31	17	2	1	36	4	50	20	0.05
Pouresmaeili et al., 2013 [28]	17	33	14	67	61	36	33	13	105	59	64	82	0.25
Duman et al., 2004 [29]	54	18	3	126	24	42	17	7	101	31	75	66	0.02
Efesoy et al., 2011 [30]	19	43	8	81	59	10	15	5	35	25	70	30	0.88
Total population											704	689	
ApaI (rs7975232 C>A)													
Sassi et al., 2015 [21]	130	143	62	403	267	90	115	26	295	167	335	231	0.23
Tanriover et al., 2010 [23]	15	29	6	59	41	22	13	15	57	43	50	50	0.00
Dundar et al., 2009 [24]	26	61	25	113	111	8	14	2	30	18	112	24	0.23
Ansari et al., 2021 [25]	120	138	32	378	202	134	107	51	375	209	292	290	0.00
Dabirnia et al., 2016 [27]	24	25	1	73	27	30	18	2	78	22	50	50	0.73
Duman et al., 2004 [29]	56	13	6	125	25	45	15	6	105	27	75	66	0.01
Total population											914	711	
TaqI (rs731236 T>C)													
Gursoy et al., 2008 [31]	20	44	6	84	56	30	29	12	89	53	70	59	0.28
Sassi et al., 2015 [21]	165	128	42	458	212	103	95	33	301	161	335	231	0.10
Uysal et al., 2008 [22]	40	46	14	126	74	54	75	17	183	109	100	146	0.24
Tanriover et al., 2010 [23]	15	29	6	59	41	25	17	8	67	33	50	50	0.10
Ansari et al., 2021 [25]	88	139	67	315	273	115	126	52	356	230	294	293	0.09
Dabirnia et al., 2016 [27]	20	24	6	64	36	16	29	5	61	39	50	50	0.12
Duman et al., 2004 [29]	23	42	10	88	62	28	23	15	79	53	75	66	0.03
Total population											974	895	

Abbreviations: HWE; Hardy–Weinberg equilibrium, M; Mutant allele, W; Wildtype allele.

**Table 4:** Summary of meta-analysis outcome that is described by the sample size, test of association, and heterogeneity.

Polymorphism ID	Sample Size			Test of Association			Heterogeneity		
	Case	Control	Studies Included	OR (95% CI)	Z	*p*-Value	X^2^	*p*-Value	I^2^ (%)
BsmI (rs1544410 G>A)									
Dominant model	704	689	7	1.27 [1.01, 1.59]	2.05	0.04 *	11.31	0.08	47%
Recessive model	704	689	7	1.04 [0.89, 1.21]	0.50	0.62	29.08	0.0001	79%
Allelic model	1408	1378	7	1.08 [0.82, 1.42]	0.53	0.60	7.37	0.29	19%
ApaI (rs7975232 C>A)									
Dominant model	914	711	6	1.03 [0.77, 1.37]	0.20	0.84	17.07	0.004	71%
Recessive model	914	711	6	0.03 [−0.02, 0.08]	1.24	0.21	4.67	0.46	0
Allelic model	1828	1422	6	0.92 [0.80, 1.07]	1.03	0.30	4.25	0.51	0
Taql (rs731236)									
Dominant model	974	895	7	1.07 [0.84, 1.38]	0.56	0.58	14.41	0.03	58%
Recessive model	974	895	7	1.13 [0.94, 1.36]	1.27	0.20	12.39	0.05	52%
Allelic model	1948	1814	7	1.12 [0.98, 1.28]	1.65	0.10	6.08	0.41	1%

## Data Availability

All data used for this analysis are presented in the manuscript.

## Supplementary Materials

The following supporting information can be downloaded at https://www.mdpi.com/article/10.3390/pathophysiology32010006/s1, Table S1: PRISMA 2020 Checklist.
