# Correction: Al-Barazenji et al. Association Between Vitamin D Receptor BsmI Polymorphism and Low Bone Mineral Density in Postmenopausal Women in the MENA Region. *Pathophysiology* 2025, *32*, 6

**DOI:** 10.3390/pathophysiology32040069

**Published:** 2025-12-03

**Authors:** Tara Al-Barazenji, Asma Allouch, Nedhal Al Husaini, Sondos Yousef, Wisam Nabeel Ibrahim, Amal Al-Haidose, Hatem Zayed, Atiyeh M. Abdallah

**Affiliations:** Department of Biomedical Sciences, College of Health Sciences, QU Health, Qatar University, Doha 2713, Qatar; ta1706200@student.qu.edu.qa (T.A.-B.); aa1602752@student.qu.edu.qa (A.A.); na2201028@student.qu.edu.qa (N.A.H.); sy1701619@student.qu.edu.qa (S.Y.); w.ibrahim@qu.edu.qa (W.N.I.); amalah@qu.edu.qa (A.A.-H.); hatem.zayed@qu.edu.qa (H.Z.)

In the original publication [1], the authors mistakenly cited a retracted article, which was reference 48. The authors have removed that reference and kept reference 47, as it sufficiently backed up their statement. With this correction, the order of some references has been adjusted accordingly. The authors state that the scientific conclusions are unaffected. This correction was approved by the Academic Editor. The original publication has also been updated.
